# Disseminated juvenile paracoccidioidomycosis: a case report and literature review

**DOI:** 10.31744/einstein_journal/2026RC1946

**Published:** 2026-01-02

**Authors:** Murilo Eduardo Soares Ribeiro, Luis Henrique Nunes de Siqueira Arouca, Julia Messina Gonzaga Ferreira, Jacyr Pasternak, Lilian Amorim Curvelo, Celso Eduardo Lourenço Matielo

**Affiliations:** 1 Hospital Israelita Albert Einstein São Paulo SP Brazil Hospital Israelita Albert Einstein, São Paulo, SP, Brazil.

**Keywords:** Paracoccidioidomycosis, Liver diseases, Mycoses, Acinetobacter infections, Endemic diseases, Tropical medicine, Child, Adolescent

## Abstract

We report a case of juvenile paracoccidioidomycosis in a previously healthy patient and aim to raise awareness of its severe and atypical clinical manifestations. A 16-year-old male student, employed in automotive cleaning services, originally from Pará and residing in Embu das Artes, São Paulo, presented with progressive lymphadenopathy, jaundice, and signs of acute liver failure. He was admitted to a tertiary hospital, where imaging and histopathological analyses confirmed the diagnosis of paracoccidioidomycosis by identifying *Paracoccidioides spp*. on lymph node biopsy using periodic acid-Schiff and Grocott's methenamine silver stains. The patient subsequently developed hepatic dysfunction, ascites, encephalopathy, and acute kidney injury. He progressed to sepsis due to *Acinetobacter baumannii* infection and required intensive care support, including broad-spectrum antimicrobial treatment, antifungal therapy, vasopressor support, and renal replacement therapy. Despite aggressive management, the patient died from septic shock and multiorgan failure. Histopathological examination confirmed disseminated fungal infection. This case highlights a rare and severe hepatic presentation of juvenile paracoccidioidomycosis and reinforces the importance of including systemic mycoses in the differential diagnosis of acute febrile illnesses accompanied by hepatosplenomegaly and lymphadenopathy in endemic regions.

## INTRODUCTION

Paracoccidioidomycosis is a systemic mycosis endemic to Central and South America, caused by dimorphic fungi of the *Paracoccidioides* genus, including the *P. brasiliensis complex* and *P. lutzii*. Although considered an uncommon disease overall, paracoccidioidomycosis remains relatively frequent in Mexico. Its distribution is particularly concentrated in Brazil, where it is endemic in states such as São Paulo, Paraná, Rio Grande do Sul, Goiás, and Rondônia. More recently, Rio de Janeiro has gained epidemiological relevance due to changes in its disease profile.^(1)^

Primary infection is typically acquired through inhalation of conidia from disturbed soil, most often during activities such as farming, gardening, or handling plant materials,^(2)^ and it is often asymptomatic.

Paracoccidioidomycosis has two clinical forms. The chronic form, responsible for approximately 80–90% of cases, predominates in rural areas and results from reactivation of latent infection. It progresses insidiously and commonly affects the lungs, mucosa, skin, adrenals, and reticuloendothelial system.^(3)^ The acute (juvenile) form, which mainly affects children and adolescents, is characterized by rapid fungal dissemination through the mononuclear phagocytic system, leading to lymphadenopathy, fever, hepatosplenomegaly, and skin lesions. Diagnosis should be considered in young patients with respiratory, mucocutaneous, or abdominal symptoms and confirmed by direct fungal identification in clinical specimens through microscopy, cytology, or histopathology.^(4)^

Herein, we report a fatal case of disseminated juvenile paracoccidioidomycosis presenting with cholestasis in a patient with no prior comorbidities. This case highlights the nonspecific yet severe nature of the disease and underscores the importance of early diagnosis in endemic, vulnerable populations.

## CASE REPORT

A previously healthy 16-year-old male student, employed part-time in automotive cleaning services, originally from Pará and residing in Embu das Artes, São Paulo, with no known comorbidities, was referred to the liver transplant intensive care unit with suspected fulminant hepatitis. He denied recent travel and reported regular exposure to soil and organic material through maintenance of enclosed microhabitats (terraria).

The patient reported insidious lymph node enlargement with progressive swelling of the submental, submandibular, and retroauricular cervical chains for approximately 1 month, followed by the onset of jaundice and exertional dyspnea over the preceding 7 days. He also experienced severe progressive right hypochondrial pain, nausea, vomiting, and dysphagia attributable to the cervical lymphadenopathy.

On admission, the patient was eupneic, normotensive, jaundiced (+2/+4), and drowsy but oriented, with tremors of the extremities. Palpable, painless lymphadenopathy measuring approximately 0.5cm was observed in the cervical, preauricular, axillary, and inguinal chains. The abdomen was tense, with massive ascites, hepatomegaly, and splenomegaly. Laboratory evaluation revealed hyperbilirubinemia (9.7mg/dL), elevated international normalized ratio (INR, 5.3), moderately increased transaminase (Aspartate Aminotransferase [AST], 133mg/dL; Alanine Aminotransferase [ALT], 153mg/dL), and elevated canalicular enzymes. Abdominal ultrasonography confirmed massive ascites, suggesting acute hepatopathy with cholestasis and prompting further liver-focused investigations.

Fulminant hepatitis was excluded due to the absence of hepatic encephalopathy. To investigate differential diagnoses—including infectious, infiltrative, neoplastic, and autoimmune causes—a lymph node biopsy was performed.

Cervical computed tomography (CT) revealed bilateral lymphadenopathy with liquefied or necrotic contents involving the cervical, retropharyngeal, retroauricular, parotid, periparotid, and occipital regions, with lymph nodes measuring up to 2.5 cm (left level IIA). A biopsy was performed for fungal investigation using periodic acid-Schiff (PAS) and Grocott's methenamine silver (GMS) staining.

Abdominal CT demonstrated hepatomegaly with lobulated contours and diffuse heterogeneous enhancement without focal nodules. Splenomegaly and nonspecific lymphadenopathy were also noted, including conglomerates measuring up to 3.3 × 2.1cm in the hepatic hilum and peripancreatic region, right inguinal lymphadenopathy measuring 2.7 × 1.1cm, and heterogeneous mesenteric lymph nodes up to 2.1cm. Magnetic resonance cholangiography revealed acute hepatopathy with ischemic injury involving the liver parenchyma and bile ducts.

Contrast-enhanced cranial CT revealed no abnormalities, and the cranioencephalic structures were within normal limits. These findings, together with the absence of meningeal signs and focal neurological deficits at presentation, made central nervous system (CNS) paracoccidioidomycosis unlikely.

**Figure 1:**
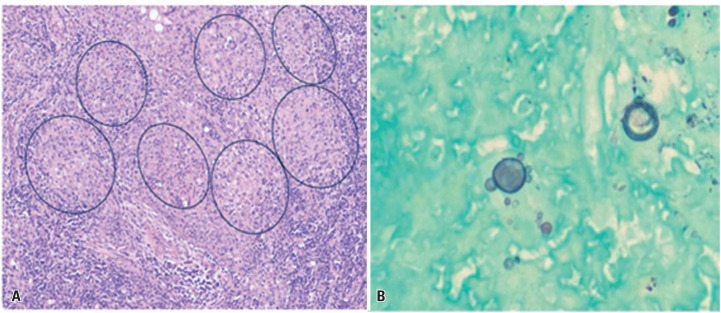
Histopathological analysis of the cervical lymph node biopsy. Periodic acid-Schiff staining showed multiple granulomas containing fungal structures (A). Grocott's methenamine silver stain revealed numerous budding yeasts with symmetrical peripheral buds forming characteristic "pilot wheel" structures, composed of a central mother cell with radially arranged daughter cells (B)

**Figure 2:**
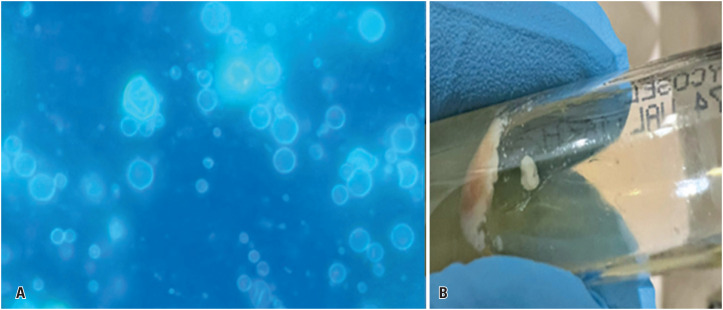
Fluorescence microscopy of a cervical lymph node biopsy showing multiple yeast-like structures with fluorophore uptake in the fungal cell wall (A). Yeast colonies grown on Sabouraud medium incubated at 35ºC (B)

A lymph node biopsy revealed marked architectural distortion due to intense neutrophilic inflammation and multiple granulomas containing fungal structures highlighted by PAS staining (Figure 1A). Numerous budding yeasts with symmetrical peripheral buds forming characteristic "pilot wheel" structures—consisting of a central mother cell with radially arranged daughter cells—were identified using GMS staining (Figure 1B). Fluorescence microscopy highlighted yeast cell walls through fluorophore labeling, and yeast colonies were observed on Sabouraud medium incubated at 35°C (Figures 2A and 2B). Antifungal therapy was promptly initiated with liposomal amphotericin B at a dose of 5 mg/kg once daily following diagnosis.

In the days following admission, the patient's clinical condition deteriorated significantly with the onset of encephalopathy, hypoglycemia, refractory ascites, and oligoanuria. Despite intensive care measures—including multiple paracenteses, transfusions, broad-spectrum antimicrobial therapy, and continuous renal replacement therapy—the patient progressed to multiorgan failure secondary to refractory septic shock, spontaneous bleeding, persistent anuria, and ultimately death. Blood and ascitic fluid cultures grew *Acinetobacter baumannii*.

This case illustrates severe hepatic involvement and systemic dissemination of the juvenile form of paracoccidioidomycosis. The clinical presentation closely mimicked fulminant hepatitis of diverse etiologies, reinforcing the importance of early differential diagnosis and consideration of systemic mycoses in patients from endemic areas.

This study was approved by the Research Ethics Committee of *Hospital Israelita Albert Einstein* (CAAE: 88557825.4.0000.0071; #7.614.561).

## DISCUSSION

Paracoccidioidomycosis is a systemic mycosis endemic to South America, particularly Brazil. The juvenile form often presents as an aggressive, disseminated disease affecting the lymph nodes, skin, and spleen. This clinical phenotype results from a complex interaction between *Paracoccidioides spp.* virulence factors and host immunogenetic susceptibility. Inhaled conidia differentiate into yeast cells in the lungs and evade innate immune responses via β- to α-glucan conversion and adhesin expression. In immunocompetent hosts, Th1/Th17 immune responses induce macrophage activation, proinflammatory cytokine production—including interferon-γ, tumor necrosis factor (TNF)-α, interleukin (IL)-17A—and organized granuloma formation.^(5)^ In contrast, in susceptible individuals, Th2 polarization and increased IL-4 production lead to impaired cell-mediated immunity, disorganized granulomas, and uncontrolled fungal proliferation.^(6)^ Genetic polymorphisms in cytokine and human leucocyte antigen genes, together with differential dendritic cell activation, further modulate disease severity.^(7)^

Hepatic involvement is common and multifactorial, occurring in approximately 30–40% of cases. It is characterized by intense inflammation, generalized lymphadenopathy, and infiltration of the mononuclear phagocytic system, leading to diffuse granulomatous infiltration of the liver parenchyma and periportal inflammation. Abdominal lymphadenopathy may further contribute by exerting extrinsic compression on the bile ducts and hepatic veins. This inflammatory cascade is likely amplified by immune-mediated mechanisms, including the release of proinflammatory cytokines such as TNF-α and IL-6.^(8)^

The nonspecific clinical manifestations of paracoccidioidomycosis, such as fever, jaundice, hepatosplenomegaly, cytopenia, and elevated transaminase levels, may mimic a wide range of infectious and hematological conditions, including acute viral hepatitis, leptospirosis, and lymphoproliferative disorders. In endemic areas, paracoccidioidomycosis should be promptly considered in the differential diagnosis of prolonged febrile syndromes associated with lymphadenopathy, as delayed recognition is associated with a significant increase in morbidity and mortality.^(2,5)^

Central nervous system involvement in paracoccidioidomycosis occurs in approximately 10–15% of cases, predominantly among rural adult males, and typically results from hematogenous spread. Neurological manifestations are diverse, with pseudotumoral lesions being the most frequently reported. Common symptoms include headaches and seizures. Diagnosis relies on CT and/or magnetic resonance imaging, which typically reveal rounded or lobulated lesions with ring or nodular enhancement and surrounding perilesional edema. Diagnostic confirmation may require a brain biopsy or the detection of specific antigens or antibodies in the cerebrospinal fluid.^(9)^

A multidisciplinary approach involving clinicians, infectious disease specialists, hepatologists, and intensivists is crucial for accurate diagnosis and timely initiation of treatment.^(10)^ This case underscores the need for heightened clinical vigilance in atypical paracoccidioidomycosis presentations, especially those with predominant hepatic involvement, and reinforces the critical role of early antifungal therapy.

## CONCLUSION

This case highlights the importance of considering paracoccidioidomycosis in the differential diagnosis of jaundice, hepatomegaly, and hepatic insufficiency, particularly in endemic regions. Early recognition and prompt multidisciplinary intervention are essential to prevent disease progression and improve patient outcomes.

## Data Availability

The underlying content is contained within the manuscript.
